# Temporal Bacteriostatic Effect and Growth Factor Loss in Equine Platelet Components and Plasma Cultured with Methicillin-Sensitive and Methicillin-Resistant *Staphylococcus aureus*: A Comparative *In Vitro* Study

**DOI:** 10.1155/2014/525826

**Published:** 2014-11-24

**Authors:** Catalina López, María E. Álvarez, Jorge U. Carmona

**Affiliations:** Grupo de Investigación Terapia Regenerativa, Departamento de Salud Animal, Universidad de Caldas, Calle 65 No. 26-10, Manizales, Colombia

## Abstract

The aims were (1) to evaluate the bacteriostatic effect of platelet-rich plasma (PRP), platelet-rich gel (PRG), leukocyte-poor plasma (LPP), leukocyte-poor gel (LPG), plasma, and heat-inactivated plasma (IP) on both methicillin-sensitive Staphylococcus aureus (MSSA) and methicillin-resistant Staphylococcus aureus (MRSA) over a period of 24 h; (2) to determine and to compare the concentrations and degradation over time of platelet factor 4 (PF-4), transforming growth factor beta 1 (TGF-*β*_1_), and platelet-derived growth factor isoform BB (PDGF-BB); and (3) to identify any correlations between MSSA and MRSA growth and either the cellular, PF-4, TGF-*β*_1_, or PDGF-BB concentrations in the blood components. PRP and its byproducts from 18 horses were obtained by the tube method. All blood components were cultured with either MSSA or MRSA. Bacterial growth, PF-4, TGF-*β*_1_, and PDGF-BB were determined at 6 h and 24 h. At six hours, bacterial growth was significantly inhibited by all blood components, with the exception of IP. MSSA was more sensitive to the treatments than MRSA. At 24 hours, bacterial growth was significantly higher in IP. MRSA bacterial growth was significantly higher in PRP, LPP, and plasma when compared to MSSA. Growth factor concentrations were not significantly affected by bacteria.

## 1. Introduction

Surgical site infections (SSI) are associated with higher morbidity, mortality, and economical losses in both human and veterinary patients.* Staphylococcus aureus* has been reported as responsible for 31–33% of the SSI in open chest procedures [1] and other major surgical procedures in humans [2]. SSI are frequent in horses after colic (linea alba infection) and orthopaedic surgery. In one study,* S. aureus* was the cause of 50% of linea alba infection in horses [3]. On the other hand,* S. aureus* has been found in 11.8% of SSI associated with orthopaedic surgical procedures in this species [4].

Although SSI are mainly produced by methicillin-sensitive* S. aureus* (MSSA), in some occasions methicillin-resistant* S. aureus* could also be present in SSI of humans [2] and horses [5]. Prophylactic and postoperative systemic antibiotherapy and the use of local antibiotics or disinfectants appear as choice treatments for the management of SSI when produced by MSSA. On the other hand, when SSI is produced by MRSA, there is not a specific antibiotic for the management of this infection [6]. In contrast, the local use of povidone-iodine, chlorhexidine [7], and even ionic silver has proved useful for the control of MRSA infections [8]. However, these substances could affect wound healing and, on the other hand, some MRSA strains are genetically resistant to these disinfectants [8, 9].

The use of platelet-rich plasma (PRP) represents a simple form of treating traumatic and chronic musculoskeletal diseases and wounds in horses and humans [10]. PRP releases transforming growth factor beta 1 (TGF-*β*_1_) and platelet-derived growth factor isoform BB (PDGF-BB) and other proteins [11–13] that have anti-inflammatory, anabolic, and angiogenic effects [10] when injected into affected tissues. Activated platelets from PRP also release proteins with microbicidal properties, such as platelet factor 4 (PF-4, CXCL4) [14]. PRP also contains mononuclear leukocytes and plasma complement [15]. The combination of all these components in PRP produces a substance with microbiostatic effects [15–17].

Several* in vitro* studies have also demonstrated the bacteriostatic effects of PRP on MRSA in horses [18] and on MSSA and other pathogenic microorganisms in humans [15–17, 19–21]. Furthermore, there are case reports showing the successful use of PRP as a treatment of human patients with chronic osteomyelitis and infected wounds [22–24]. These encouraging effects have also been observed in a rabbit model of MRSA osteomyelitis [25].

*In vitro* studies of either equine or human PRP have focussed on whether the antimicrobial effect of PRP is dependent on leukocyte activity and whether these substances can inhibit bacterial growth over time [15–18, 21]. However, these studies have not evaluated whether these bacteria induce the degradation or loss of growth factors, such as TGF-*β*_1_ and PDGF-BB, and whether there are differences between MSSA and MRSA associated with their susceptibility to PRP or their capability for denaturing these growth factors. Furthermore, to the best of the authors' knowledge, no information is available on the possible role of PF-4 as a microbicidal or microbiostatic protein in equine or human PRP [18, 19, 26].

The aims of this study were (1) to evaluate the antibacterial effects of PRP, platelet-rich gel—PRG—(PRP activated with calcium gluconate), and other equine blood components, including leukocyte-poor plasma (LPP), leukocyte-poor gel (LPG), plasma, and heat-inactivated plasma (IP), on both MSSA and MRSA over a period of 24 h; (2) to determine and to compare the concentrations and degradation over time of PF-4, TGF-*β*_1_, and PDGF-BB in all equine blood components cultured with both bacteria; and (3) to establish possible correlations between MSSA and MRSA growth and either the cellular counts (platelets and leukocytes) or the concentrations of PF-4, TGF-*β*_1_, or PDGF-BB of each blood component.

## 2. Material and Methods

This study was approved by the Ethical Committee for Animal Experimentation of the authors' institution.

### 2.1. Horses

Eighteen clinically healthy horses (geldings) with a median age of 12 years (range: 4–18) were included. The animals were from two farms with similar environmental and husbandry conditions. The owners of the horses were informed of the nature of the research and signed the appropriate authorization.

### 2.2. Preparation of Equine PRP and the Other Blood Components

**Figure 1:**
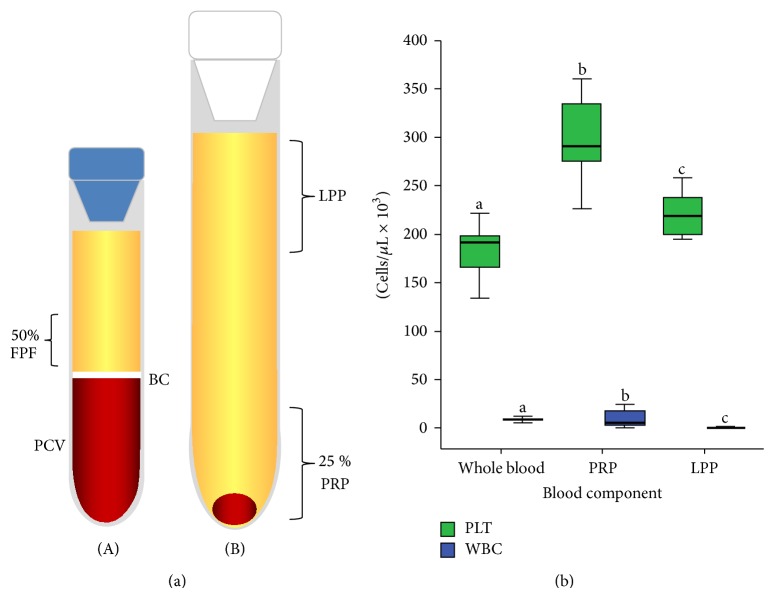
(a) Graphic illustrating the plasma fractions obtained from the tube-method protocol. The left tube (A) contains the first plasma fraction (50%) (FPF) obtained by the single centrifugation tube method. The right tube (B) contains platelet-rich plasma (PRP) and leukocyte-poor plasma (LPP). BC: buffy coat. PCV: packed cell volume. (b) Graphic illustrating the platelet (PLT) and leukocyte (WBC) mean (±SD) concentrations in whole blood, PRP, and LPP. ^a-b^Different lowercase letters represent significant differences for the count of each cell in every blood component (*P* > 0.001).

Whole blood was aseptically drawn from the jugular vein using a 23-G butterfly catheter (Blood Collection Set, Vacutainer, Franklin Lakes, NJ, USA) and was placed in 4.5 mL 3.2% sodium citrate tubes (BD Vacutainer). PRP was prepared using the tube method [27]. Blood was centrifuged at 120 ×g for 5 min. The first supernatant plasma fraction (50%), adjacent to the buffy coat, was obtained and centrifuged again at 240 ×g for 5 min, and the PRP and leukocyte-poor plasma (LPP) fractions were obtained (Figure 1(a)). Plasma was obtained from citrated blood centrifuged at 3500 ×g for 5 min. IP was prepared from plasma treated with heat at 65°C for 30 min for denaturing its complement.

### 2.3. Study Design and* In Vitro* Antibacterial Assay

The study included evaluations of PRP, PRG (activated in a 1 : 10 ratio with 9.3 mg/mL calcium gluconate), leukocyte-poor plasma activated with calcium gluconate (LPG), plasma, and IP. All liquid blood components, except IP, were evaluated before calcium gluconate activation for their platelet and leukocyte counts with a haematology impedance analyser (Celltac-*α* MEK 6450, Nihon Kohden, Tokyo, Japan).

Blood components were assigned to eight groups: PRP, PRG, LPP, LPG, plasma, IP, positive control group (PCG) (Müller-Hinton broth with bacteria), and negative control group. An aliquot (2.5 mL) from each group was mixed with 16 *μ*L of a suspension of either MSSA (ATCC 29213, OxoidCulti-Loops, KS, USA) or MRSA (ATCC 43300 [C9022L], OxoidCulti-Loops, KS, USA) to obtain a final concentration of 1 × 10^6^ colony-forming units (CFU)/mL. The samples from each group were serially diluted 1 : 10 (1 : 10, 1 : 100, 1 : 1000, 1 : 10000, and 1 : 100000), and 10 *μ*L of each dilution was plated on 5% sheep-blood agar plates and incubated at 37°C for 6 and 24 h. The CFU on each plate were counted by eye. The highest growth recorded for any sample was 300 CFU/plate for all dilutions. The number of CFU/mL was determined with the formula CFU/mL = CFU/plate × (1/0.01 mL aliquot plated) × dilution factor. The supernatant of each sample was aliquoted and stored at −80°C for later determination of PF-4 and growth factor concentrations.

### 2.4. Determination of PF-4, TGF-*β*_1_, and PDGF-BB Concentrations with Enzyme-Linked Immunosorbent Assays (ELISAs)

The PF-4, TGF-*β*_1_, and, PDGF-BB concentrations from each blood component cultured with each bacterium were determined in duplicate by sandwich ELISAs developed with commercial antibodies for human PF-4 (human CXCL4/PF-4, DY795; R&D Systems, Inc., Minneapolis, MN, USA), TGF-*β*_1_ (human TGF-*β*_1_, DY240E; R&D Systems, Inc.), [28] and PDGF-BB (human PDGF-BB, DY220; R&D Systems, Inc.) [29]. The thresholds of detection were 15.6 pg/mL for PF-4, 31.2 pg/mL for TGF-*β*_1_, and 31.2 pg/mL for PDGF-BB. The ELISAs were performed according to the manufacturer's instructions. Readings were made at 450 nm [13].

A 2.5 mL sample of PRP and LPP from each horse was incubated at 37°C for 15 minutes with 250 *μ*L of a solution containing 0.5% of a nonionic detergent (NID) (TritonX100, Sigma-Aldrich Co. LLC., MO, USA). Both blood components treated with NID were used as a positive control of PF-4 and GF release [30, 31]. It is important to consider that TGF-*β*_1_ and PDGF-BB have been measured in equine PRP by using human ELISA antibodies [13, 27, 32].

### 2.5. Statistical Analysis

The statistical analysis was performed with the software IBM SPSS 19.0 (SPSS Inc., Chicago, IL, USA). A Shapiro-Wilk test was used to assess the fit of data set to a normal distribution (goodness of fit). Both platelet and leukocyte counts in whole blood, PRP, and LPP presented a normal distribution (*P* > 0.05). CFU/mL, PF-4, TGF-*β*_1_, and PDGF-BB concentrations showed a nonparametric distribution (*P* > 0.05), even after attempting logarithmic, root square, and rank transformations.

The platelet and leukocyte counts in whole blood, PRP, and LPP are presented as the mean (±SD) and analysed by one-way ANOVA followed by Turkey's* post hoc* test. The antibacterial effect and the PF-4 and growth factor concentrations of each treatment group at 6 and 24 h are presented as a median (interquartile range) and evaluated independently with a Kruskal-Wallis test followed by a Mann-Whitney *U post hoc* test. Wilcoxon and Mann-Whitney *U* tests were used for comparing the effect of each treatment on bacterial growth, PF-4, TGF-*β*_1_, and PDGF-BB concentrations at 6 and 24 h. A Spearman test (*r*_*s*_) was used to identify possible correlations of either MSSA or MRSA bacterial growth (CFU/mL) at any time with PLT and WBC counts or with PF-4, TGF-*β*_1_, and PDGF-BB concentrations in blood components. A *P* value > 0.05 was accepted as statistically significant for all tests.

## 3. Results and Discussion

### 3.1. Haematological Values

Platelet and leukocyte counts differed significantly between whole blood, PRP, and LPP (*P* > 0.001) (Figure 1(b)). PRP and LPP presented a platelet enrichment of 1.64X and 1.21X, respectively, in comparison with the original platelet counts in whole blood. On the other hand, PRP presented a 2X leukocyte concentration in comparison with the leukocyte concentration in whole blood. LPP presented an extreme leukoreduction (−93X) in comparison with the basal leukocyte concentration in whole blood.

### 3.2. Bacteriostatic Effects of Blood Components over Time

At 6 hours, bacterial growth of both bacteria was significantly inhibited by all blood components, with the exception of IP, in comparison to PCG. MSSA was more sensitive to the treatments than MRSA. PRP presented a better bacteriostatic effect against MRSA in comparison with LPG and plasma. LPG and plasma were significantly less effective for controlling MRSA growth when compared to MSSA (Table 1).

At 24 hours, bacterial growth for both bacteria was significantly higher for PCG followed by IP. MSSA growth was significantly inhibited in order of importance by PRP, plasma, LPP, LPG, and PRG. Plasma, LPG, and LPP showed a significantly better bacteriostatic effect against MSSA when compared to PRG. In general, MRSA bacterial growth was similar in PRP, PRG, and LPP when compared to IP. It was observed that LPG and plasma presented a significant inhibition of MRSA growth when compared to IP. MRSA bacterial growth was significantly higher in PRP, LPP, and plasma when compared to MSSA (Table 1).

### 3.3. PF-4 Concentration over Time in Each Blood Component Cultured with Either MSSA or MRSA

At 6 h, the PF-4 concentration was significantly higher in IP cultured with MSSA when compared to plasma and LPG cultured with the same bacterium and the lysates of PRP (PRP + NID) and LPP (LPP + NID). Blood products cultured with MRSA, particularly, PRG, LPP, and IP, presented significantly higher concentrations of this protein when compared to plasma. IP cultured with MRSA had a significantly higher PF-4 concentration when compared to PRP and LPG (Table 2).

At 24 h, PRP, LPP, plasma, and IP cultured with MSSA presented higher PF-4 concentrations when compared to the lysates of PRP and LPP. All the blood components cultured with MRSA presented higher PF-4 concentrations when compared to the lysates of PRP and LPP. The PF-4 concentration was significantly higher in IP cultured with MRSA when compared to IP cultured with MSSA (Table 3).

### 3.4. TGF-*β*_1_ Concentrations over Time in Each Blood Component Cultured with Either MSSA or MRSA

PRP + NID presented significantly higher concentrations of TGF-*β*_1_ in comparison with the other blood components indistinctly of the cultured bacteria. At 6 hours, plasma and IP cultured independently with every bacterium had a significantly lower TGF-*β*_1_ concentration when compared to PRG, LPP, LPG, and both PRP and LPP lysates. TGF-*β*_1_ presented a significantly higher concentration in PRP-cultured MSSA when compared to PRG, LPG, plasma, and IP cultured with the same bacterium. PRG cultured with MRSA presented a significantly higher TGF-*β*_1_ when compared to the same blood component cultured with MSSA (Table 4).

At 24 h, a similar trend in the TGF-*β*_1_ concentrations was observed, as noted at 6 hours, for all blood components evaluated. However, no differences were noted between every blood component independently cultured with a specific bacterium (Table 5).

### 3.5. PDGF-BB Concentrations over Time in Each Blood Component Cultured with Either MSSA or MRSA

At 6 hours, PDGF-BB presented a significantly lower concentration in IP when compared to the other blood components, independent of the cultured bacterium. Conversely, PRP indistinctly of the cultured bacterium presented the highest PDGF-BB concentration in comparison to IP, plasma, and LPP + NID. However, the PDGF-BB concentration was similar between PRP, PRG, LPP, and LPG independently of the cultured bacterium (Table 6). At 24 hours, PDGF-BB concentrations in the groups evaluated presented a similar trend to that observed at 6 hours. However, IP cultured with MSSA presented significantly lower PDGF-BB concentrations in comparison to the same blood component cultured with MRSA (Table 7).

### 3.6. Correlations

No correlations were observed between either MSSA or MRSA counts (CFU/mL) and WBC counts, platelet counts, and PF-4, TGF-*β*_1_, and PDGF-BB concentrations in the blood components evaluated over time.

There are several proposals intended to classify a plethora of platelet-rich plasmas used in human and equine practice [10]. One of them had classified the PRPs obtained with anticoagulants in both pure-platelet-rich plasma (P-PRP) and leukocyte platelet-rich plasma (L-PRP). Equine P-PRP displays slightly higher platelet counts (1.3–4-fold) and leukocyte (WBC) counts (0.5–2-fold) than whole blood. Equine L-PRP has increased platelet (5-fold) and leukocyte (3-fold or more) counts when compared to whole blood [10]. Platelet concentrates evaluated in this study can be classified as P-PRPs or leukoreduced platelet concentrates [13, 33].

The methodology used in this study is different from the bacteriologic methods used in former human [17] and equine [18] research. In previous studies, the platelet concentrates were cultured with bacteria in Müller-Hinton broth. In the present study, the culture broth was not added because the authors wanted to determine if the culture media could affect the growth of the bacteria by diluting the PRP components. This situation is particularly important since PRP is used clinically alone and not with Müller-Hinton broth. The present research was designed to know whether PRP could inhibit* S. aureus* growth when it is contaminated accidentally during its processing.

It is paramount to mention that* in vitro* PRP displays a short temporal bacteriostatic effect against* S. aureus*; this obligates the use of a strict aseptic protocol to obtain the blood and then to prepare the PRP, independently of the technique used for its processing [34]. On the other hand, it is possible to state that although the results from the present study and other results previously reported in other research [18] are encouraging, there is not enough information for supporting the clinical use of this substance in equine SSI. However, the present research opens new avenues for evaluating the effect of PRP in equine models of wound infection or in clinical trials that include horses with natural SSI.

Results from the present research possibly indicate that the* in vitro* bacteriostatic effect of equine PRP could be mainly mediated by plasma complement [15]. However, it is also possible that for* in vivo* conditions the bacteriostatic effect of PRP may be mediated by other biological mechanisms that include leukocytes and microbicidal proteins, such as PF-4 [14]. It is important to note that this protein was not correlated with the inhibition of bacterial growth in this research. These results could be attributed to the fact that a human anti-PF-4 antibody was used to detect this protein in the equine blood components. To date, the degree of identity between human and equine PF-4 has not been published, although one study demonstrated that this protein in equines can be measured by ELISA using a human anti-PF-4 antibody [35]. However, the PF-4 results of the present study should be accepted with caution until the equine PF-4 protein is fully characterized at the molecular level.

One of the most important findings of this study was that LPG and plasma showed a better bacteriostatic effect against MSSA in comparison to MRSA at 6 hours and then at 24 hours; a significant bacteriostatic effect of PRP was noted against MSSA but not against MRSA. The same results were also evident for LPP and plasma. It is possible that there is a genetic basis to the mechanism of resistance to PRP in MRSA. However, this hypothesis should be tested in future research.

In clinical conditions, infected wounds (e.g., SSI) present an adverse environment for an adequate healing process. Wound bacteria induce severe inflammation that in some cases could develop to a chronic state. Local factors such as metalloproteinase production and the release of bacterial proteases can denature key growth factors directly implicated in wound healing [36]. The* in vitro* results from the present study are encouraging because both bacteria possibly were not able to denature or induce loss of the measured growth factors during the first 24 hours and, importantly, did not affect the metabolic activity of the platelet concentrates evaluated.

These findings may suggest that these blood products not only have a bacteriostatic effect, but also have a detrimental action on the metabolic activity of the bacteria that prevents the loss of proteins such as TGF-*β*_1_ and PDGF-BB. It is important to consider that, in some cases, chronic wounds in humans (and even in horses) require treatment with very expensive recombinant growth factors for improving wound healing [37]. However, this treatment is contraindicated when the wound is severely infected, since bacterial proteases and host metalloproteinases produce rapid growth factor degradation [38]. Results from this study may indicate that PRP and its byproducts could be used for the treatment of these wounds because they contain higher concentrations of autologous growth factors in addition to an important antibacterial effect. However, this hypothesis needs* in vivo* investigation.

## 4. Conclusions

(1) Plasma complement could be implicated in the bacteriostatic effect of equine PRP and the other blood components. (2) MSSA was more sensitive to equine PRP when compared to MRSA. (3) All the blood products evaluated, and even IP, could have produced a metabolic impairment in both bacteria, which prevented the growth factor loss. (4) In general, PRP had the better characteristics because it was bacteriostatic and presented a higher concentration of growth factors. (5)* In vivo* studies are necessary for recommending the use of PRP and its byproducts as a treatment of SSI contaminated with* S. aureus* in horses.

## Figures and Tables

**Table 1:** Median (interquartile ranges (IR)) for both methicillin-sensitive *Staphylococcus aureus* (MSSA) and methicillin-resistant *S. aureus* (MRSA) (CFU/mL × 10^8^) (1 : 100 × 10^3^ dilution) for every blood component over time.

Blood components	MSSA	MRSA	MSSA	MRSA
	6 hours		24 hours	
Positive control group (PCG)	280 (330)^a^	425 (1007.5)^a^	2400 (1595)^a^	2580 (1135)^a^
Platelet-rich plasma (PRP)	0.0 (2.5)^b^	0.0 (0.0)^b,c,d^	55 (95)^b,e^	570 (640)^B^
Platelet-rich gel (PRG)	0.0 (0.0)^b^	0.0 (10)^b^	265 (455)^b,d,f^	410 (470)
Leukocyte-poor plasma (LPP)	0.0 (10)^b^	0.0 (10)^b,d^	80 (112.5)^b^	535 (285)^B^
Leukocyte-poor gel (LPG)	0.0 (0)^b^	5.0 (30)^b,A^	140 (450)^b^	350 (462)^b^
Plasma	0.0 (10)^b^	10 (22.5)^b,A^	75 (75)^b^	305 (395)^b,C^
Heat inactivated plasma (IP)	15 (52.5)	45 (122)	580 (610)	645 (260)

^a–f^Different lowercase letters represent significant differences between each treatment group at each specific time. ^a^Significantly different from all treatments (*P* > 0.001). ^b^Significantly different from IP (*P* > 0.001). ^c^Significantly different from LPG (*P* > 0.05). ^d^Significantly different from plasma (*P* > 0.05). ^e^Significantly different from PRG (*P* > 0.05). ^f^Significantly different from LPP (*P* > 0.05). ^A–C^Different capital letters represent significant differences in the same row for a specific treatment group in relation to both bacteria during a specific period of time. ^A^ *P* > 0.05. ^B^ *P* > 0.001. ^C^ *P* > 0.01.

**Table 2:** PF-4 (pg/mL) concentration in every blood component cultured with either MSSA or MRSA at 6 hours.

Blood components	MSSA	MRSA
Platelet-rich plasma (PRP)	2331 (3561)	2012 (2310)^a^
Platelet-rich gel (PRG)	2703 (2549)	2991 (4992)^c^
Leukocyte-poor plasma (LPP)	2066 (3187)	3553 (3187)^c^
Leukocyte-poor gel (LPG)	1216 (2772)^a^	844 (5704)^a^
Plasma	685 (2558)^a^	594.5 (2172.5)
Heat-inactivated plasma (IP)	4087 (8054)^b^	2756 (3957)^c^
PRP + NID	1322.5 (1842.75)	1322.5 (1842.75)
LPP + NID	1322 (2103)	1322 (2103)

^a-b^Lowercase letters represent significant differences in the same column. ^a^Significantly different from IP (*P* > 0.05). ^b^Significantly different from PRP + NID and LPP + NID (*P* > 0.01). ^c^Significantly different from plasma (*P* > 0.01). NID: nonionic detergent.

**Table 3:** PF-4 (pg/mL) concentration in every blood component cultured with either MSSA or MRSA at 24 hours.

Blood components	MSSA	MRSA
Platelet-rich plasma (PRP)	3872 (6851.50)^a^	2278 (2814)^a^
Platelet-rich gel (PRG)	897 (3915.5)	2491 (2124)^a^
Leukocyte-poor plasma (LPP)	4671 (4621)^a^	2916 (4275)^a^
Leukocyte-poor gel (LPG)	1668 (2389)	2119 (3588)^a^
Plasma	2809.5 (3163.5)^a^	4084 (6033)^a^
Heat-inactivated plasma (IP)	3022 (3086)^a^	4615 (9188)^a∗^
PRP + NID	1322.5 (1842.75)	1322.5 (1842.75)
LPP + NID	1322 (2103)	1322 (2103)

^a^Lowercase letters represent significant differences in the same column. ^a^Significantly different from PRP + NID and LPP + NID (*P* > 0.05). ^*^Significantly different concentrations for both bacteria in IP (*P* > 0.05). NID: nonionic detergent.

**Table 4:** TGF-*β*_1_ (pg/mL) concentration in every blood component cultured with either MSSA or MRSA at 6 hours.

Blood components	MSSA	MRSA
Platelet-rich plasma (PRP)	4038 (3440)^a^	4700 (2934)^g^
Platelet-rich gel (PRG)	3064 (1184)^b^	3608 (1512)^h∗^
Leukocyte-poor plasma (LPP)	3904 (1596)^c^	3088 (1904)^h,i^
Leukocyte-poor gel (LPG)	3116 (1469)^c,d^	2838 (1626)^c,d^
Plasma	2032 (938)^e^	1722 (948)^e^
Heat-inactivated plasma (IP)	1590 (1085)^e^	1460 (903)^e^
PRP + NID	6688 (3548)^f^	6688 (3548)^f^
LPP + NID	4400 (1362)	4400 (1362)

^a-b^Lowercase letters represent significant differences in the same column. ^a^Significantly different from PRG (*P* > 0.05), LPG (*P* > 0.01), plasma (*P* > 0.001), IP (*P* > 0.001), and PRP + NID (*P* > 0.05). ^b^Significantly different from LPG (*P* > 0.01), plasma (*P* > 0.001), IP (*P* > 0.001), PRP + NID (*P* > 0.001), and LPP + NID (*P* > 0.001). ^c^Significantly different from plasma (*P* > 0.001), IP (*P* > 0.001), and PRP + NID (*P* > 0.001). ^d^Significantly different from LPP + NID (*P* > 0.001). ^e^Significantly different from PRP + NID and LPP + NID (*P* > 0.001). ^f^Significantly different from LPP + NID (*P* > 0.01). ^g^Significantly different from LPG (*P* > 0.01), plasma (*P* > 0.001), IP (*P* > 0.001), and PRP + NID (*P* > 0.05). ^h^Significantly different from plasma (*P* > 0.001), IP (*P* > 0.001), and PRP + NID (*P* > 0.01). ^i^Significantly different from LPP + NID (*P* > 0.01). ^*^Significantly different concentrations for both bacteria in PRG (*P* > 0.05). NID: nonionic detergent.

**Table 5:** TGF-*β*_1_ (pg/mL) concentration in every blood component cultured with either MSSA or MRSA at 24 hours.

Blood components	MSSA	MRSA
Platelet-rich plasma (PRP)	4544 (3916)^a^	4032 (2116)^g^
Platelet-rich gel (PRG)	4352 (2314)^b^	3664 (1338)^b^
Leukocyte-poor plasma (LPP)	3452 (1402)^c^	3048 (1944)^c^
Leukocyte-poor gel (LPG)	3248 (1240)^c^	3356 (1666)^c^
Plasma	1588 (1448)^d^	2030 (1332)^d^
Heat-inactivated plasma (IP)	1524 (1164)^e^	1660 (1096)^e^
PRP + NID	6688 (3548)^f^	6688 (3548)^f^
LPP + NID	4400 (1362)	4400 (1362)

^a-b^Lowercase letters represent significant differences in the same column. ^a^Significantly different from LPP, LPG, plasma, and IP (*P* > 0.05) (0.001). ^b^Significantly different from LPG (*P* > 0.05), plasma, IP (*P* > 0.001), PRP + NID, and LPP + NID (*P* > 0.05) (0.001). ^c^Significantly different from IP, PRP + NID (*P* > 0.001), and LPP + NID (*P* > 0.01). ^d^Significantly different from PRP + NID and LPP + NID (*P* > 0.001). ^e^Significantly different from PRP + NID and LPP + NID (*P* > 0.001). ^f^Significantly different from LPP + NID (*P* > 0.01). ^g^Significantly different from plasma, IP, and PRP + NID (*P* > 0.001). NID: nonionic detergent.

**Table 6:** PDGF-BB (pg/mL) concentration in every blood component cultured with either MSSA or MRSA at 6 hours.

Blood components	MSSA	MRSA
Platelet-rich plasma (PRP)	2690.5 (4842)^a^	2977 (2629.75)^a^
Platelet-rich gel (PRG)	1998 (2222)^b^	2123.1 (1474)^b^
Leukocyte-poor plasma (LPP)	2200 (3762)^c^	4405 (3230)^c^
Leukocyte-poor gel (LPG)	2106 (5787.25)^c^	3230.5 (3389.5)^c^
Plasma	185.5 (720.5)^d^	182,5 (518.75)^e^
Heat-inactivated plasma (IP)	0 (70.75)^e^	0 (235.5)^e^
PRP + NID	2389.5 (732.5)^f^	2389.5 (732.5)^f^
LPP + NID	1755 (806.5)	1755 (806.5)

^a-b^Lowercase letters represent significant differences in the same column. ^a^Significantly different from plasma, IP (*P* > 0.001), and LPP + NID (*P* > 0.01). ^b^Significantly different from plasma and IP (*P *>0.001). ^c^Significantly different from plasma and IP (*P* > 0.001). ^d^Significantly different from IP (*P* > 0.01), PRP + NID, and LPP + NID (*P* > 0.001). ^e^Significantly different from PRP + NID and LPP + NID (*P* > 0.001). ^f^Significantly different from LPP + NID (*P* > 0.01). NID: nonionic detergent.

**Table 7:** PDGF-BB (pg/mL) concentration in every blood component cultured with either MSSA or MRSA at 24 hours.

Blood components	MSSA	MRSA
Platelet-rich plasma (PRP)	2330 (942.5)^a^	2080 (1568.5)^b^
Platelet-rich gel (PRG)	1723 (3305)^b^	1904 (6577.5)^b^
Leukocyte-poor plasma (LPP)	2163 (3520)^b^	1746 (1613)^b^
Leukocyte-poor gel (LPG)	2225 (2913.5)^b^	3690 (3674)^b^
Plasma	88 (616.5)^c^	330.5 (894)^c^
Heat-inactivated plasma (IP)	0 (0.5)^d^	13 (183.5)^d∗^
PRP + NID	2389.5 (732.5)^e^	2389.5 (732.5)^e^
LPP + NID	1755 (806.5)	1755 (806.5)

^a^Significantly different from plasma, IP (0.001), and LPP + NID (*P* > 0.05). ^b^Significantly different from plasma and IP (*P* > 0.001). ^c^Significantly different from IP (*P* > 0.05), PRP + NID, and LPP + NID (*P* > 0.001). ^d^Significantly different from PRP + NID and LPP + NID (*P* > 0.001). ^e^Significantly different from LPP + NID (*P* > 0.01). ^*^Significantly different concentrations for both bacteria in IP (*P* > 0.05). NID: nonionic detergent.
